# Blue and green shade nets differentially regulate organic acid accumulation in citrus fruit

**DOI:** 10.3389/fpls.2026.1969799

**Published:** 2026-10-14

**Authors:** Abdelmonem Elshahat, Yong-Zhong Liu, Essam Elatafi, Abdullah Alebidi, Abebe Assefa Gobena, Hai-Qiang Liu, Yin Luo, Basma Elhendawy, Rohoma Tahir, Sami Ur Rehman, Abdul Baess Keyhani, Rashid S. Al-Obeed, Mahmoud Abdel-Sattar

**Affiliations:** 1 National Key Laboratory for Germplasm Innovation and Utilization of Horticultural Crops, College of Horticulture and Forestry Sciences, Huazhong Agricultural University, Wuhan, Hubei, China; 2 Department of Horticulture, Faculty of Agriculture, Kafrelsheikh University, Kafr El-Sheikh, Egypt; 3 Key Laboratory of Genetics and Fruit Development, College of Horticulture, Nanjing Agricultural University, Nanjing, China; 4 Department of Pomology, Faculty of Agriculture, Mansoura University, Mansoura, Egypt; 5 Department of Plant Production, College of Food and Agriculture Sciences, King Saud University, Riyadh, Saudi Arabia

**Keywords:** citrus sinensis, colored shade nets, fruit acidity, organic acid metabolism, vacuolar acidification

## Abstract

Colored shade nets modify orchard microclimate and may influence citrus fruit metabolism and quality. We evaluated their effects on organic acid accumulation in ‘Newhall’ sweet orange (*Citrus sinensis*) over two consecutive growing seasons. In the first season, red, yellow, green, and blue nets at 40% and 60% shading were compared with open-field conditions; green 40% and blue 60%, which showed contrasting acidity responses, were evaluated further in the second season. Shade nets reduced photosynthetically active radiation, light intensity, and canopy temperature, increased relative humidity, maintained PSII efficiency, and generally lowered stress-related indicators. Blue 60% maintained the highest citric acid, malic acid, and titratable acidity, whereas green 40% produced the lowest acidity. These patterns were associated with distinct transcriptional responses: blue 60% showed sustained higher *CsPH8* expression and stage-specific increases in *CS2* and *CS1*, while green 40% showed higher expression of several organic-acid degradation-related genes, including *ACO*, *ACL3*, *FUM*, and *NAD-ME*. Correlation analysis further linked lower radiation and temperature, higher relative humidity, and improved PSII performance with greater organic acid accumulation and expression of acid synthesis- and storage-related genes. Collectively, the findings suggest that blue 60% shading is associated with improved organic acid retention through coordinated microclimatic, physiological, and transcriptional responses, supporting its potential for maintaining citrus fruit acidity under warm, high-radiation conditions.

## 1 Introduction

Organic acid accumulation driven mainly by citrate and malate is a defining component of citrus flavor because it determines fruit acidity and strongly influences the sugar/acid balance during development and ripening (Etienne et al., 2013; Batista-Silva et al., 2018). At the cellular level, final acidity reflects a dynamic balance among organic acid synthesis, metabolic turnover, and vacuolar compartmentation, with these processes occurring across multiple organelles and being tightly regulated over time (Alam et al., 2022; Mao et al., 2024; Guo et al., 2025). In this context, genes associated with citrate synthesis (*CS1/CS2*) and downstream tricarboxylic acid (TCA) cycle-related steps (**Figure 1**) involved in citrate/malate interconversion and consumption (*ACOs, ACLs, MDHs, FUM, NAD-ME*) have been widely linked to developmental changes in fruit acidity and cultivar-to-cultivar variation (Batista-Silva et al., 2018; Sadka et al., 2019). Acid regulation involves not only metabolism but also vacuolar acidification and transport, which enhance organic-acid sequestration and retention by generating a strong proton gradient (Huang et al., 2021). In citrus fruits, functional evidence has identified *CsCIT1* as a vacuolar citrate/H^+^ symporter involved in citrate storage dynamics (Shimada et al., 2006), specific transporters such as *ALMT*-family proteins for malate as well, and multiple studies have highlighted vacuolar proton-pumping components as key determinants of citrus vacuolar pH and acid accumulation (Shi et al., 2019; Strazzer et al., 2019). The transcriptional regulation of these genes is highly responsive to both developmental cues and environmental signals, positioning them as key targets for quality manipulation. Organic acid concentrations in citrus fruit are therefore dynamic throughout development and ripening, as the balance among synthesis, degradation, and vacuolar storage changes over time. These processes are also responsive to environmental conditions, including light and temperature, which can alter the trajectory of acid accumulation and loss and consequently final fruit acidity (Etienne et al., 2013; Batista-Silva et al., 2018; Sadka et al., 2019). Thus, shade-induced changes in the fruit microenvironment may influence organic acid dynamics during development.

**Figure 1:**
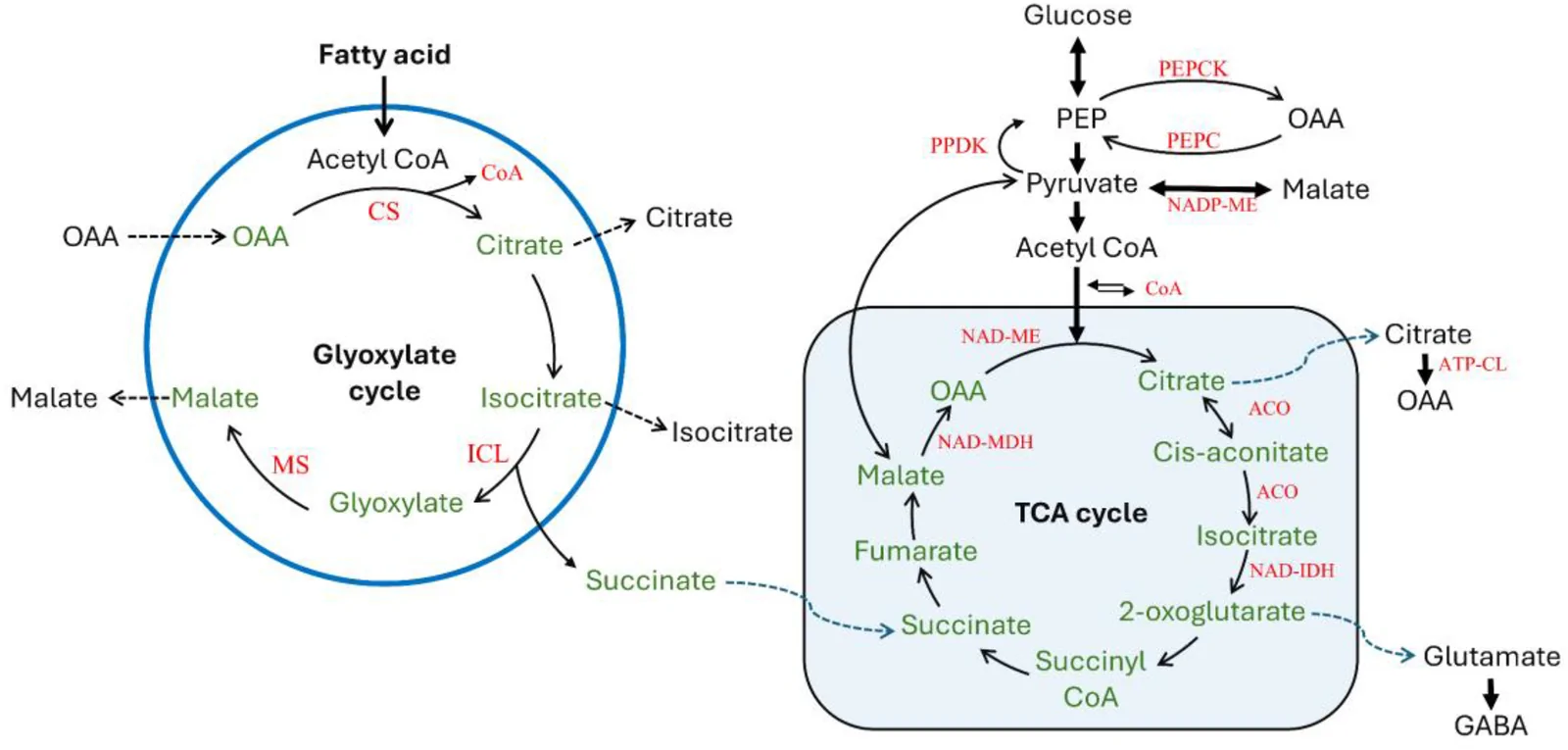
Metabolic pathways of citric acid and malic acid in fruit. TCA, tricarboxylic acid cycle; *ACO*, aconitase; ATP-CL, ATP-citrate lyase; *CS*, citrate synthase; *ICL*, isocitrate lyase; *MS*, malate synthase; *NAD-MDH, NAD*-malate dehydrogenase; *NAD-ME, NAD*-malic enzyme; *NAD-IDH, NAD*-isocitrate dehydrogenase; *NADP-ME, NADP*-malic enzyme; OAA, oxaloacetate; PDH, pyruvate dehydrogenase; PEPC, phosphoenolpyruvate carboxylase; PEPCK, phosphoenolpyruvate carboxykinase; PPDK, pyruvate orthophosphate dikinase.

Colored shade nets have therefore emerged as a promising orchard-level strategy to steer fruit quality because they simultaneously modify light quantity, spectral composition, and the tree microclimate (Shahak, 2008; Ilić and Fallik, 2017). By filtering radiation and reducing heat load, shade nets can lower canopy temperature and alter humidity and light distribution within the canopy, thereby affecting photosynthesis, stress responses, and downstream metabolic regulation (Shahak, 2008; Wachsmann et al., 2014). These spectrally modified nets filter sunlight, altering the red:far-red and blue: green light ratios reaching the canopy, inducing specific physiological responses while also providing a buffer against environmental extremes (Sheng et al., 2017; Książek, 2023; Su et al., 2025). Changes in photosynthetic performance are often captured through chlorophyll fluorescence, where *Fv/Fm* is widely used as an indicator of PSII functional status under field stressors such as high light and heat (Maxwell and Johnson, 2000; Elshahat et al., 2026). Beyond physiology, a growing body of work supports the idea that colored shade nets can translate microclimate and light signals into shifts in ripening-related pathways, ultimately affecting quality traits in fruit-tree systems (Mditshwa et al., 2019; Ju et al., 2023; Zhang et al., 2023; Zaman et al., 2025). **Figure 1** provides a helpful illustration of this principle and motivates testing whether a similar microclimate PSII mechanism could be linked to the regulation of organic-acid accumulation through changes in the expression of acidity-related genes (Etienne et al., 2013; Huang et al., 2021). Therefore, shade nets serve as integrated environmental modulators that can influence gene expression networks related to primary metabolism, providing a new method for regulating the biochemical composition of fruits, including their acidity (Stamps, 2009; Bastías et al., 2012).

Despite evidence supporting the benefits of shading for fruit quality, the relative contributions of spectral properties, shading intensity, and associated microclimatic changes to organic acid metabolism in citrus fruit remain unclear. Accordingly, this study investigates how colored shade nets influence organic acid accumulation in citrus fruit across two consecutive growing seasons. By integrating microclimate monitoring, physiological stress profiling, organic acid measurements, and gene expression analysis, the study evaluates associations among shade-induced environmental changes, fruit physiological responses, and acidity-related transcription patterns. This approach provides a framework for assessing colored shade nets as a potential strategy for maintaining citrus fruit acidity under warm, high-radiation conditions.

## 2 Materials and methods

### 2.1 Plant materials and treatments

The experiment was conducted during two successive growing seasons (2023–2024 and 2024-2025) at the Citrus Research Orchard, Huazhong Agricultural University, Wuhan, Hubei Province, China. The site has a subtropical monsoon climate, characterized by hot, humid summers and cold, damp winters, with occasional snowfall and high annual rainfall (Wuhan Meteorological Bureau, 2022) and trees were planted in alluvial soil. Eight-year-old ‘Newhall’ sweet orange (*Citrus sinensis*) trees grafted onto trifoliate orange (*Poncirus trifoliata*) were used. The field experiment was arranged as a randomized complete block design (RCBD) with three blocks (biological replicates). In the first season, nine treatments were evaluated: the open-field control and eight shade-net combinations consisting of red, yellow, green, and blue nets at 40% and 60% shading. Each treatment was represented by three trees within each block; therefore, each treatment included nine trees in total, and the first-season experiment comprised 81 trees (9 treatments x 3 replicates x 3 trees). Each treatment-by-block group of three trees constituted the field experimental unit, and individual trees served as sampling units within each replicate. In the second season, three treatments were evaluated (open-field control, green 40%, and blue 60%) using the same replication structure, giving 27 trees in total (3 treatments x 3 replicates x 3 trees). The same trees assigned to the control, green 40%, and blue 60% treatments in the first season were retained for the second season. During the second season, green 40% and blue 60% were selected from the first-season treatments because they showed the strongest contrasting organic acid responses, with blue 60% producing the highest and green 40% the lowest citric and malic acid concentrations (**Figure 2A**). The treatment differences were supported by the statistical analysis shown in **Figure 2A**. Thus, treatment selection was based primarily on the contrasting organic acid response rather than on physiological performance; physiological measurements were subsequently used to help interpret the observed responses. The open-field treatment was retained as the control (**Figure 3**). The shading treatment was applied for approximately five months, from mid-June to mid-November, covering the post-fruit-set fruit-development period through harvest. Shading was initiated two weeks after full fruit set, thereby deliberately excluding the flowering and initial fruit-set stages. This duration was selected to allow the effects of shading to be evaluated throughout fruit development and ripening until harvest without directly affecting flowering or fruit set. The shade nets were placed on a steel tube structure and metal wires, placed 4.5 m above the ground and 1.5 m above the tree canopy. To minimize exposure to low-angle sunlight during early morning and late afternoon, vertical shade netting was installed on both sides of the tree rows. Fruit sampling differed between seasons according to the experimental objective. In the first season, which served as the broad screening experiment, six uniform fruits per tree were harvested from different canopy positions at the final sampling stage for all nine treatments. In the second season, the same control, green 40%, and blue 60% trees were retained and sampled at 30, 60, 90, 120, and 150 days after shading (DAS) to characterize developmental changes in organic-acid accumulation. At each sampling date, all nine trees per treatment were sampled, and six fruits were collected from each tree (54 fruits per treatment per date). Fruits were selected for uniform size and similar external development. Sampling was conducted on the same dates for all three treatments, thereby matching fruit by chronological stage after shading while applying comparable external developmental criteria. All collected fruit samples were immediately frozen in liquid nitrogen, ground to powder, and stored at -80 °C for subsequent biochemical and gene-expression analyses.

**Figure 2:**
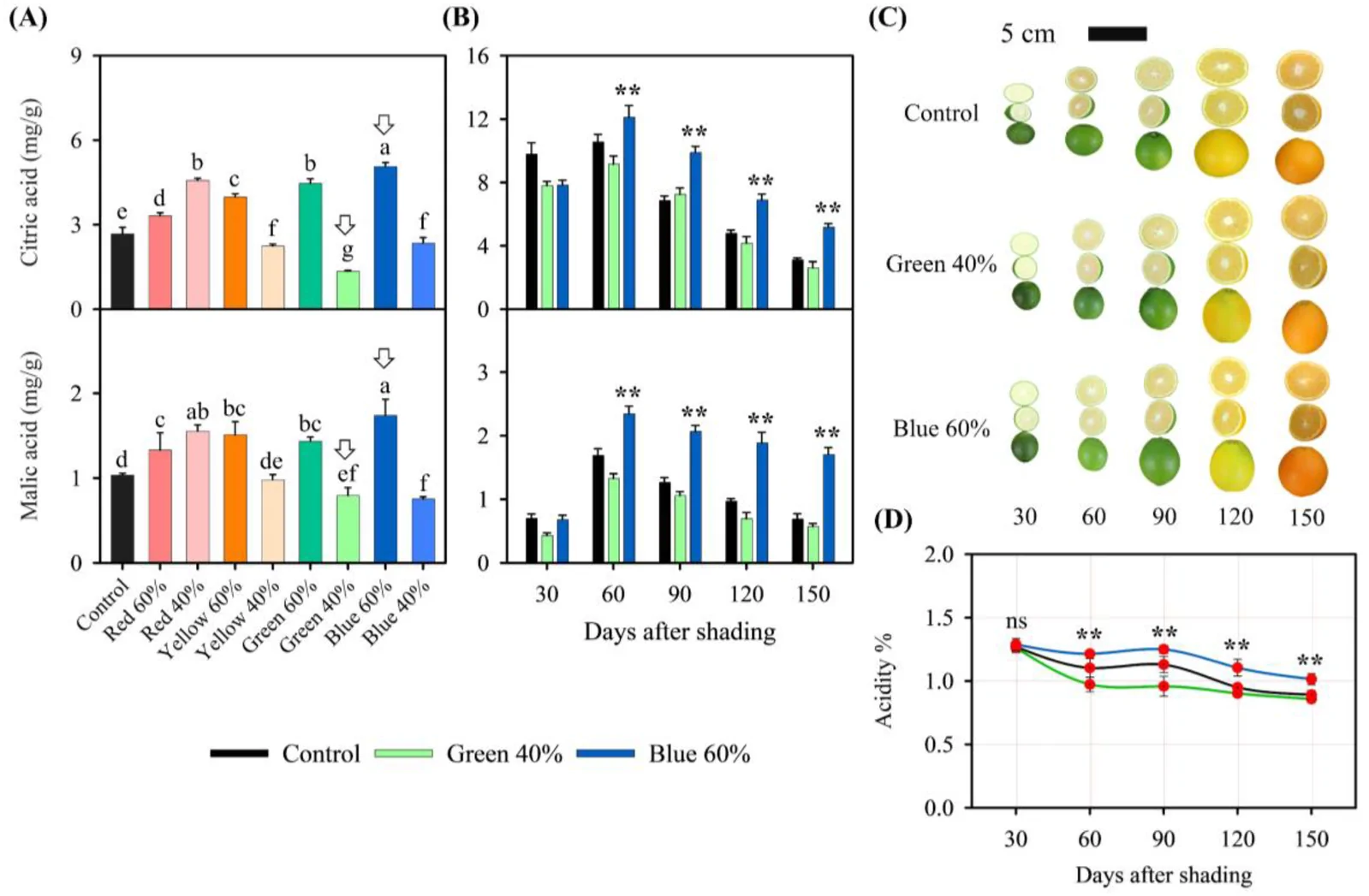
Influence of color shade-nets on organic acids content during the field experiments of 2023–2024 and 2024–2025 respectively **(A, B)**, the fruit sampling **(C)**, and titratable acidity **(D)** during 2024–2025 season. Values are the mean ± SD. A column with different lowercase letters indicates a statistically significant difference (P< 0.05) when the data were performed by variance analysis, followed by Duncan’s multiple range tests. Asterisks (* or **) indicate significant differences at P< 0.05 or P< 0.01 between treatments.

**Figure 3:**
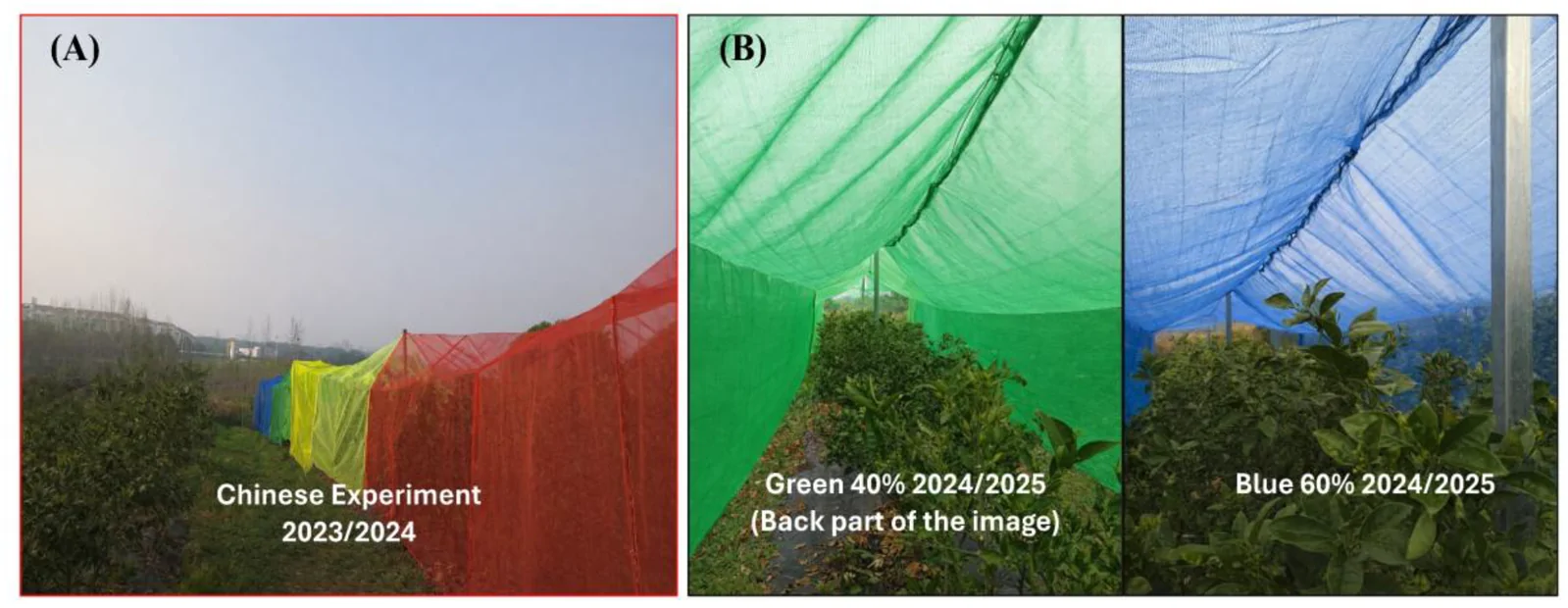
The field experiment site during the 2023–2024 season **(A)** and the 2024–2025 season **(B)**, Huazhong Agricultural University, Wuhan, Hubei Province, China.

The difference in sampling strategy between seasons was intentional. The first season was used to screen a broad set of shade-net color and intensity combinations and identify treatments with contrasting acidity responses. The second season focused on the control, green 40%, and blue 60% treatments and used multiple sampling dates to resolve the developmental trajectory of organic-acid and transcriptional responses.

### 2.2 Environmental and photometric measurements

In this study, we continuously monitored temperature and relative humidity (RH) using 12 Hygrometer Thermometer Temperature Dew Point & RH devices from Reed Instruments under both shaded and open-field conditions, temperature and relative humidity (RH) were continuously monitored in both growing seasons using the same instrumentation and measurement approach, with local meteorological data used as a reference. To assess inter-season comparability, open-field temperature and RH were used as common environmental references between years. The corresponding control means are reported in **Table 1** and indicate broadly comparable mean temperature and RH during the two experimental periods, while normal interannual environmental variation could not be excluded. During the shading experiment, photosynthetic active radiation (PAR) and light intensity (LUX) were measured at different times of the day and at various positions within the canopy using a BTs256-EF Lux meter. Average measurements were calculated for different-colored shade nets and the open field after the shading period. The efficiency of Photosystem II (*Fv/Fm*) was determined using a handheld chlorophyll fluorometer (OS30P+). Measurements were performed on healthy, mature, fully developed spring-shoot leaves located approximately 3–5 leaves from the shoot apex; these leaves were selected to ensure sufficient maturity for stable photosynthetic function. Three trees from each treatment were used for the measurements. Leaves were dark-adapted for at least 15 min before measurement. *Fv/Fm* was assessed after one month of shading, at the midpoint, and at the end of the shading period. At each measurement occasion, readings were taken twice daily, at 11:00 AM and 4:00 PM. Measurements for all treatments were conducted on the same dates and within the same time windows under the same prevailing orchard weather conditions, while treatment-specific microclimatic conditions generated by the shade nets were maintained as part of the experimental treatments.

**Table 1:** Average temperatures and relative humidity under colored shade nets and control during the field experiments of 2023–2024 and 2024-2025.

Seasons	2023/2024									2024/2025		
Treatments	Control	Red 60%	Red 40%	Yellow 60%	Yellow 40%	Green 60%	Green 40%	Blue 60%	Blue 40%	Control	Green 40%	Blue 60%
Temperature (°C)	28.02	22.61	25.20	22.73	25.13	22.59	25.23	22.68	25.25	28.55	25.48	23.05
RH (%)	68.91	75.70	72.82	75.88	72.88	75.88	72.99	75.33	72.80	69.14	73.19	75.57

### 2.3 Organic acid determination and titratable acidity assessment

To estimate organic acid content, the protocol described by Bartolozzi et al. (1997) was used. In detail, about 3.0 g of freeze-dried powder fruit pulp sample was dissolved in 15 mL of 80% methanol (v/v). The mixture was incubated in a water bath at 70 °C for 30 min, then subjected to ultrasonic treatment for 45 min, and finally centrifuged at 3000 rpm for 20 min. After centrifugation, the supernatant was collected in a 50 mL round-bottom flask, and the above extraction was repeated twice. Afterward, 1 mL of internal standard solution (2.5% methyl-D-glucopyranoside, 2.5% phenyl-D-glucopyranoside, and 10% acetone) was added, and the final volume was achieved with 80% methanol. The solution was mixed, and an aliquot of 1.5 mL was transferred to a 2 mL Eppendorf tube, followed by centrifugation at 12000 rpm for 30 min. After centrifugation, an aliquot of 0.5 mL of supernatant was sampled into a new 2 mL Eppendorf tube and evaporated at 60 °C for about 2 h. The residue was dissolved in 0.8 mL of hydroxylamine hydrochloride solution in pyridine (20 mg/mL), incubated at 75 °C for 1 h, and then cooled to room temperature. Subsequently, 0.4 mL of hexamethyldisilane and 0.2 mL of trimethylchlorosilane were added, and the mixture was incubated at 70 °C for 2 h. The samples were then kept overnight at room temperature, followed by centrifugation at 12000 rpm for 30 min. The supernatant was filtered through a 0.22 μm filter and analyzed by gas chromatography using an Agilent 7890B gas chromatograph (Agilent, USA) with a flame ionization detector. A nonpolar HP-5 MS phenyl-methyl-siloxane column with 0.25 μm film thickness (30.0 m long × 0.32 mm i.e., USA) was used for separation. The front inlet and detector temperatures were set to 270 °C and 300 °C, respectively. The flow rates of H_2_, N_2_, and air were 25, 30, and 400 mL/min, respectively. The sample volume injected was 1 μL, and the split ratio was 30:1. The temperature program started at 130 °C and was set to rise to 198 °C at 6 °C/min, then at 0.5 °C/min to 205 °C, and then at 20 °C/min to the final temperature of 280 °C, at which it was maintained for 4 min. The organic acid content was determined with standard curves, and all standards were purchased from Sigma (St. Louis, MO, USA). TA was assessed through titration with sodium hydroxide (NaOH, 0.1 M) and phenolphthalein (1%) as a pH indicator.


$$\text{Acidity }(\%)=\frac{\text{Volume of NaOH}*0.1*0.064*100}{\text{Sample volume}}$$

### 2.4 Antioxidant enzymes, proline, and abscisic acid assessment

Antioxidant enzyme activities were measured using commercial microplate assays. Superoxide dismutase (SOD) was quantified via the WST-8 method at 450 nm based on inhibition of formazan production. Catalase (CAT) activity was determined by monitoring H_2_O_2_ breakdown using a chromogenic probe at 510 nm. Peroxidase (POD) activity was assessed by guaiacol oxidation at 470 nm. Tissue samples were homogenized in cold extraction buffer, centrifuged, and the supernatants were analyzed. Activities were reported per unit mass, with sample volumes adjusted to remain within the linear detection range. All measurements were performed in triplicate. Free proline was quantified using a colorimetric microplate assay based on the acidic ninhydrin reaction. Fruit tissues were homogenized in sulfosalicylic acid buffer, incubated at 90 °C, and centrifuged. At 95 °C for 30 minutes, the supernatant was mixed with acidic ninhydrin and glacial acetic acid. The absorbance was read at 520 nm. A proline standard curve (0-20 µg/mL) was used to measure the amount. Sample volumes were adjusted to maintain linearity, and extractions were repeated three times. Abscisic acid (ABA) was quantified following a modified procedure (Gobena et al., 2025). Pulp samples (0.1 g) were mixed with isopropanol/water/HCl in glass centrifuge tubes. After vortexing, an internal standard was added, and samples were agitated for 1 h. Dichloromethane was added, followed by another 1 h of agitation and centrifugation. The lower phase was isolated, concentrated, and reconstituted. All steps were performed in the dark at 4 °C. ABA levels were analyzed by HPLC, with three replicates per treatment.

### 2.5 Candidate gene selection and sequence analysis

Gene sequences were retrieved and validated using BLAST analysis via three citrus genomic databases: the Orange Genome Annotation Project (Huazhong Agricultural University; http://citrus.hzau.edu.cn), NCBI (https://www.ncbi.nlm.nih.gov), and Phytozome (https://phytozome-next.jgi.doe.gov/pz/portal.html). The search employed an E-value cutoff of E-30 and the BLOSUM62 comparison matrix, selecting the highest-scoring matches for downstream analysis. Subsequently, gene-specific primers, including actin reference primers (**Supplementary Table S1**), were designed using Primer Premier 5.0 (Premier Biosoft, USA) based on the confirmed genomic sequences.

### 2.6 RNA extraction and quantitative real-time PCR

Total RNA was extracted from fruit samples using the method previously described by Liu et al. (2006). cDNA was synthesized from 1 μg of high-quality total RNA using the Transcript One-step gDNA Removal and cDNA Synthesis SuperMix (Transgene Biotech, Beijing, China). Quantitative real-time PCR (qRT-PCR) was performed in a 10-μl reaction volume with Hieff qPCR SYBR Green Master Mix (YEASEN, Shanghai, China) on a QuantStudio™ 6 Flex Real-Time PCR System (Thermo Fisher Scientific, USA), following the manufacturer’s protocol. The Ct values were normalized to the *actin* gene as referenced in prior studies (Ma et al., 2019). Data analysis was conducted using the Livak method (Livak and Schmittgen, 2001). All primer sequences are provided in **Supplementary Table S1**.

### 2.7 Pearson correlation analysis

Correlations among all measured variables were evaluated using Pearson’s correlation coefficient (R) with the ChiPlot online platform (https://www.chiplot.online). The magnitude of each linear association was interpreted using the R value, where coefficients approaching 1 indicate stronger positive correlations and those approaching -1 indicate stronger negative correlations (Schulze et al., 2012).

### 2.8 Statistical analysis

Statistical analysis utilized CoStat software and one-way ANOVA to test the null hypothesis of equal treatment variances. Treatment means were assessed using Duncan’s multiple-range test, with significance levels set at *P* < 0.05 and *P* < 0.01, indicated by unique lowercase letters or asterisks. Data are reported as mean ± standard deviation (SD) from at least three biological replicates. Gene expression patterns were hierarchically clustered using ChiPlot (https://www.chiplot.online).

## 3 Results

### 3.1 Comparison of environmental conditions

The two-year field experiment (2023-2025) evaluated the effects of colored shade nets (red, yellow, green, and blue) with varying shading intensities (40% and 60%) on microclimate parameters, and the experimental data demonstrated that all shading net treatments exerted significant microclimatic modifications compared to the unshaded treatment (control) across both shading seasons (**Table 1**; **Figure 4**). Compared to the open field, all shade nets significantly reduced mean ambient temperature (**Figures 4A, B**), with shade nets 60% showed a higher temperature reduction rate compared to control. The shade nets provided a 40% reduction in intermediate temperature. In general, there was a clear inverse relationship between shading percentage and temperature, with shading at 60% intensity producing the most significant mean temperature depression of 5.1-6.0 °C relative to the control, while shading at 40% yielded intermediate reductions (2.7-3.4 °C) (**Table 1**).

**Figure 4:**
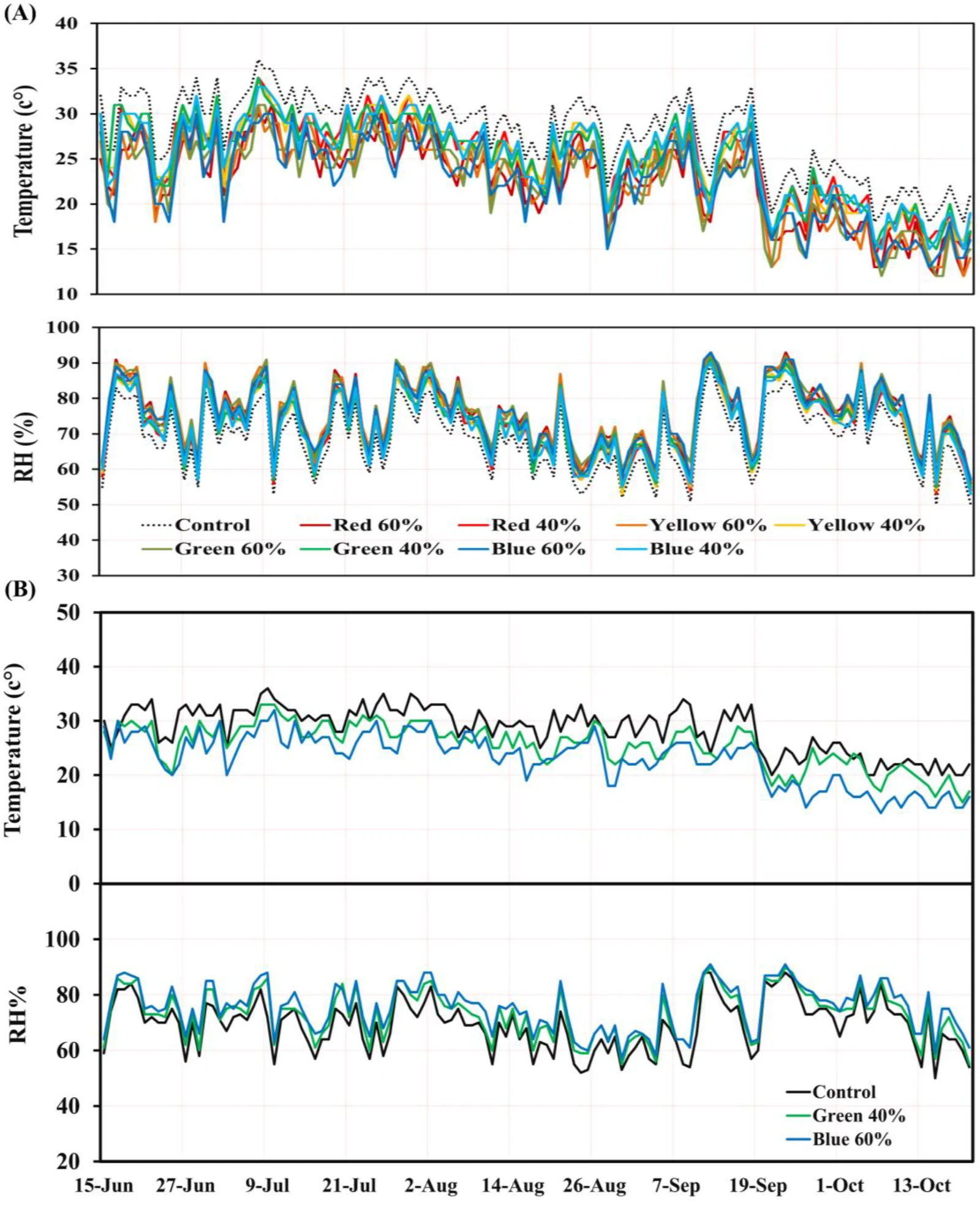
Change of temperatures and relative humidity under colored shade nets and control during the field experiments of 2023-2024 **(A)** and 2024-2025 **(B)**.

Conversely, RH increased systematically under shading, with shade nets 60% maintained higher RH than shading nets 40% and the control (**Figures 4A, B**); a positive correlation was found between shading intensity and RH; shading 60% elevated RH by 6.0-8.6% while shading 40% increased it by 3.9-5.1% compared to control (**Table 1**). Notably, although shading intensity had a significant effect, net color exerted no statistically significant effect on temperature or RH, indicating that microclimate modulation was primarily governed by shading percentage rather than spectral properties.

The open-field control showed similar mean environmental conditions between seasons: mean temperature was 28.02 °C in 2023–2024 and 28.55 °C in 2024-2025, while mean RH was 68.91% and 69.14%, respectively (**Table 1**), indicating broadly comparable baseline thermal and humidity conditions.

### 3.2 Comparison of photosynthetic active radiation, light intensity (LUX), and photosystem II efficiency (Fv/Fm)

In addition to its effect on the microclimate surrounding citrus trees, shading nets had a clear impact on photometric measurements (**Figure 5**). The results from the first season revealed that PAR and LUX were highest in the open field compared to all shading treatments (**Figure 5A**). Among the shading treatments, those with a shade ratio 60% showed lower PAR and LUX rates than those with a shade ratio 40%. The results in the second season were in the same direction, as PAR and LUX rates were higher in the open field than in the two shading treatments (green 60% and red 60%) (**Figure 5B**). In addition to the above, we found that during the first season, all shade net treatments maintained, or slightly improved, PSII efficiency compared to the open-field control, with shading nets 60% (particularly green and blue) showing the most consistent positive effects (**Figure 5A**). The nets 60% enhanced PSII efficiency by 5-8% over control conditions, while shading nets 40% showed more minor improvements (2-3%). During the second season, when testing only green 60% and red 60% nets compared to the open field, both maintained PSII efficiency observed in the previous season, as in the control treatment (**Figure 5B**).

**Figure 5:**
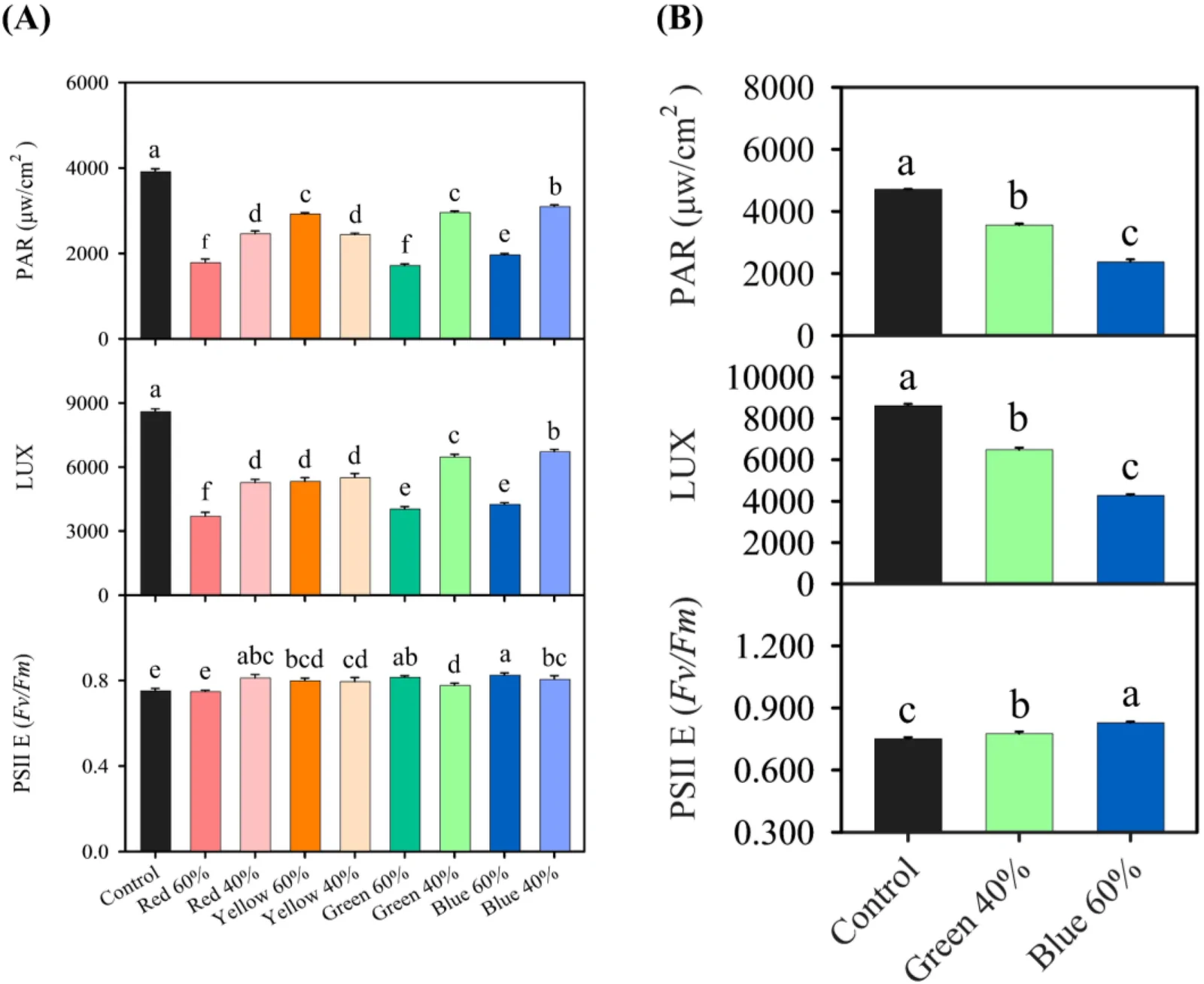
Average of Photosynthetic Active Radiation, LUX, and photosystem II efficiency under colored shade nets and the open field (Control) during the field experiments of 2023-2024 **(A)** and 2024-2025 **(B)**. Values are the mean ± SD. A column with different lowercase letters indicates a statistically significant difference (*P* < 0.05) when the data were performed by variance analysis, followed by Duncan’s multiple range tests.

### 3.3 Comparison of organic acid content and titratable acidity

In the 2023–2024 comparison across multiple net types for organic acid (**Figure 2A**), the blue 60% shade net consistently produced the highest citric and malic acid levels. In contrast, the green 40% net produced the lowest, and the remaining colors tended to fall between these extremes (**Figure 2A**).

During second season it was demonstrated that shading influenced the trajectory of acidification during development (**Figures 2B, C**). Both acids generally declined with time after shading, as expected during ripening due to dilution from growth and metabolic consumption. However, the blue 60% shade net maintained significantly higher citric and malic acid concentrations from 60 DAS onward, while the green 40% showed the lowest values, with the control intermediate. The same ranking was reflected in titratable acidity (**Figure 2D**), which decreased over time in all treatments but remained highest under blue 60%, significantly diverging from the other treatments after the early stage.

### 3.4 Comparison of antioxidant enzymes, proline, and abscisic acid

The open-field condition imposed greater stress than shaded environments. Catalase (CAT) activity was notably highest in the control group across all sampling intervals (30, 90, and 150 DAS), while shading treatments, particularly the green 40% net, resulted in a significant reduction in CAT activity (**Figure 6A**). Peroxidase (POD) exhibited a similar pattern, with greater activity noted in the control at multiple points, despite a transient increase observed with blue 60% net around 90 DAS (**Figure 6B**). In contrast, superoxide dismutase (SOD) activity remained non-significant across sampling dates (**Figure 6C**). It is clear that the subsequent hydrogen peroxide removal mechanisms (CAT and POD) were more influenced by the microclimatic variations induced by shading. Non-enzymatic stress indicators, such as proline concentration, confirmed these findings, being higher in the open field throughout the sampled periods and significantly lower under both shade nets (**Figure 6D**). Furthermore, abscisic acid (ABA) levels were consistently elevated in the control group and showed a significant reduction under shaded conditions (**Figure 6E**). This overall analysis underscores the differential stress responses of plants subjected to varying light conditions, elucidating the biochemical indicators influenced by environmental shading.

**Figure 6:**
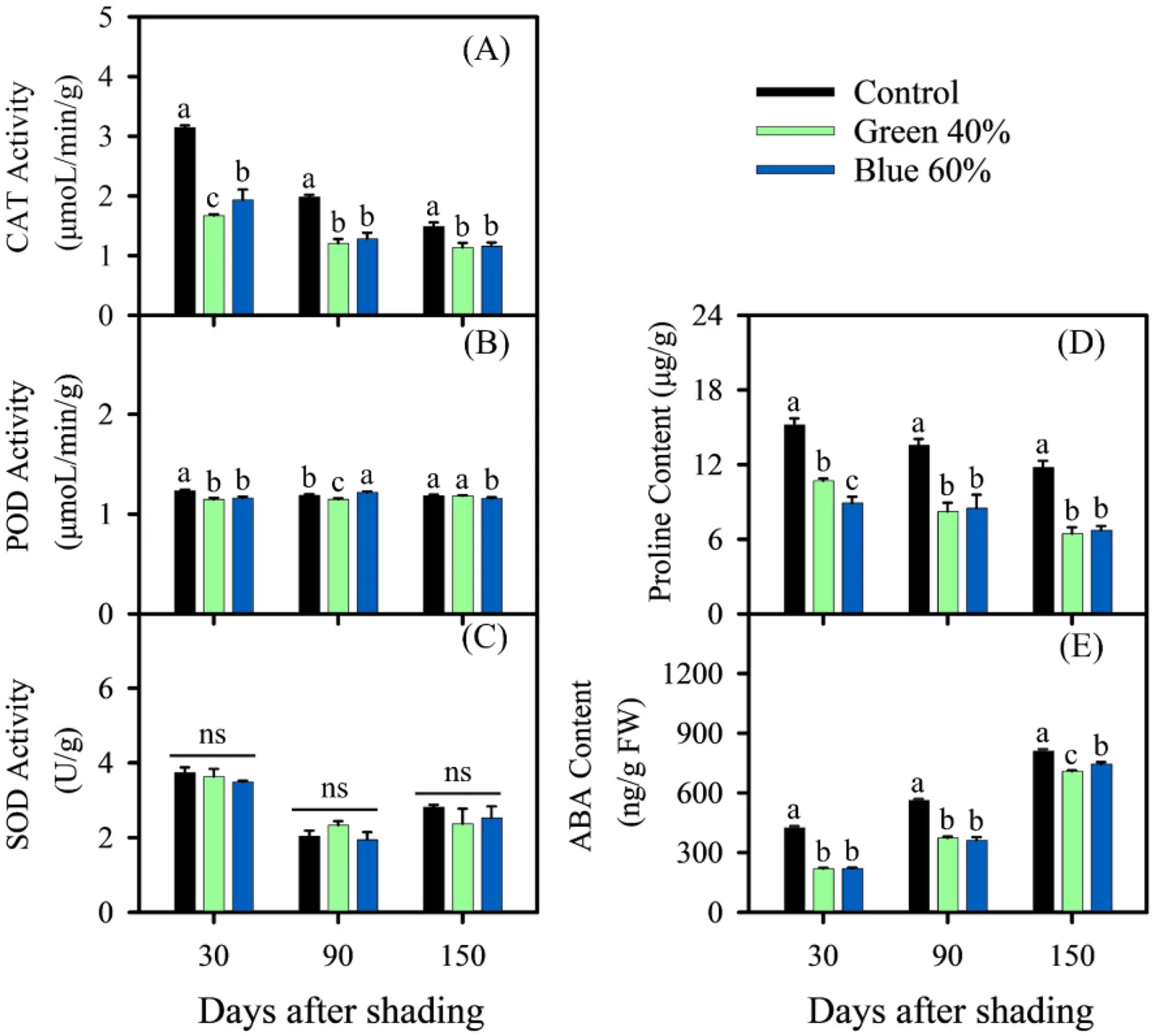
Average of antioxidant enzymes **(A–C)**, proline **(D)**, and abscisic acid **(E)** under colored shade nets and the open field (control) during the field experiments of 2024-2025. Values are the mean ± SD. A column with different lowercase letters indicates a statistically significant difference (P< 0.05) when the data were analyzed by analysis of variance, followed by Duncan’s multiple range tests.

### 3.5 Transcript analysis of citrate-malate synthesis-related genes

In the 2023–2024 season (**Figure 7A**), the shading response was mainly expressed as a treatment-dependent shift in citrate-synthesis genes. *CS1* was significantly lower under green 40% than under the control, while blue 60% maintained *CS1* at a level comparable to the control; *CS2* was upregulated considerably under blue 60% relative to both the control and green 40%; in contrast, *NAD-MDH* and *MS* showed no significant differences. In the 2024–2025 season (**Figure 7B**), the response was developmental stage-dependent: at 30 DAS, green 40% transiently increased *CS1* and *NAD-MDH*; by 90 DAS, blue 60% shade net showed a significant peak in *CS2*, consistent with enhanced citrate synthesis capacity mid-season, and by 150 DAS, blue 60% shade net exhibited significantly higher *CS1*, implying a late-season maintenance of citrate formation potential during the period when organic acids typically decline, while *MS* remained largely unchanged across treatments.

**Figure 7:**
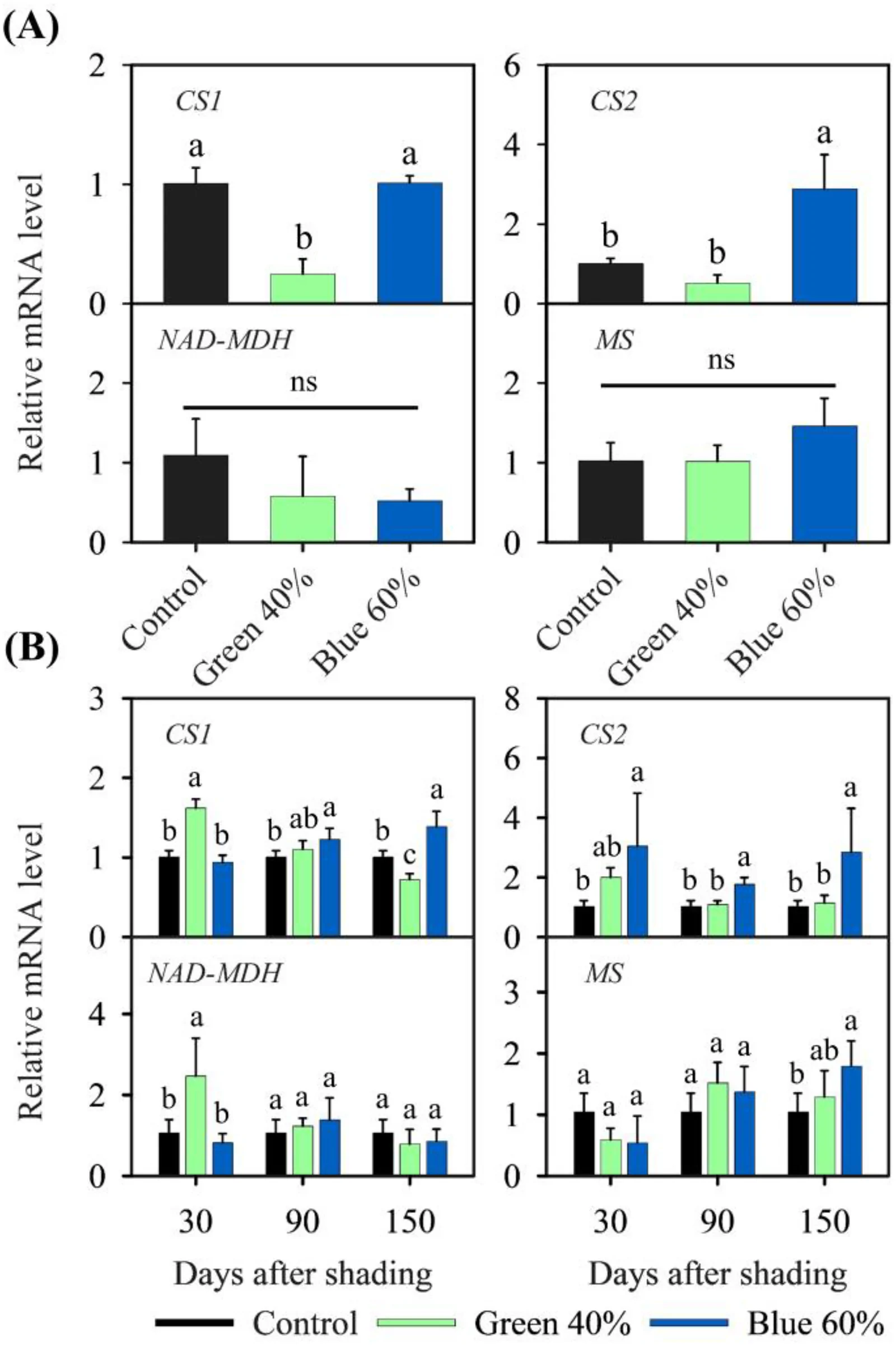
Transcript comparison of citrate-malate synthesis-related genes under color shading nets during the field experiments of 2023–2024 at harvest **(A)** and 2024–2025 at three different stages after shading **(B)**. Values are the mean ± SD. A column with different lowercase letters indicates a statistically significant difference (P< 0.05) when the data were analyzed by analysis of variance, followed by Duncan’s multiple range tests.

### 3.6 Transcript analysis of citrate-malate degradation-related genes

In the 2023–2024 season, the two major upstream aconitase transcripts (*ACO1* and *ACO2*) were not significantly different between the two shaded treatments, but *ACO3* was markedly lower under shading, especially under blue 60%.

Several citrate-cleavage transcripts (*ACL1* and *ACL2*) were also reduced under shade (lowest in green 40%), whereas *ACL3* showed the opposite pattern and was strongly induced, particularly under green 40%, implying a shift in which *ACL* isoform contributes to citrate turnover under altered light. Downstream malate-associated genes also differed among treatments, with *FUM* and *NAD-ME* showing the highest transcript abundance under green 40%. The higher *NAD-ME* expression was associated with lower malic acid accumulation, suggesting a potential contribution to malate utilization. However, as enzyme activity and metabolic flux were not measured, this relationship should be interpreted as an association rather than direct evidence of increased malate degradation. Blue 60% showed lower expression of these degradation-related transcripts, consistent with greater acid retention (**Figure 8A**). In the 2024–2025 season, treatment effects were most pronounced early and mid-development. At 30 DAS, green 40% significantly upregulated multiple genes, including *ACO1*/*ACO2*/*ACO3*, *ACL1*/*ACL2*, and *MDH2*. At 90–150 days, the pattern became more selective: blue 60% showed significant increases in some aconitase transcripts (*ACO1*/*ACO2*/*ACO3*) at mid/later stages, while green 40% maintained strong induction of other downstream genes (notably *FUM* and *ACL3* at later time points). Overall, the transcript data indicate that green 40% is associated with earlier and broader expression of organic-acid degradation-related genes, whereas blue 60% shows a more limited or temporally shifted transcriptional response (**Figure 8B**).

**Figure 8:**
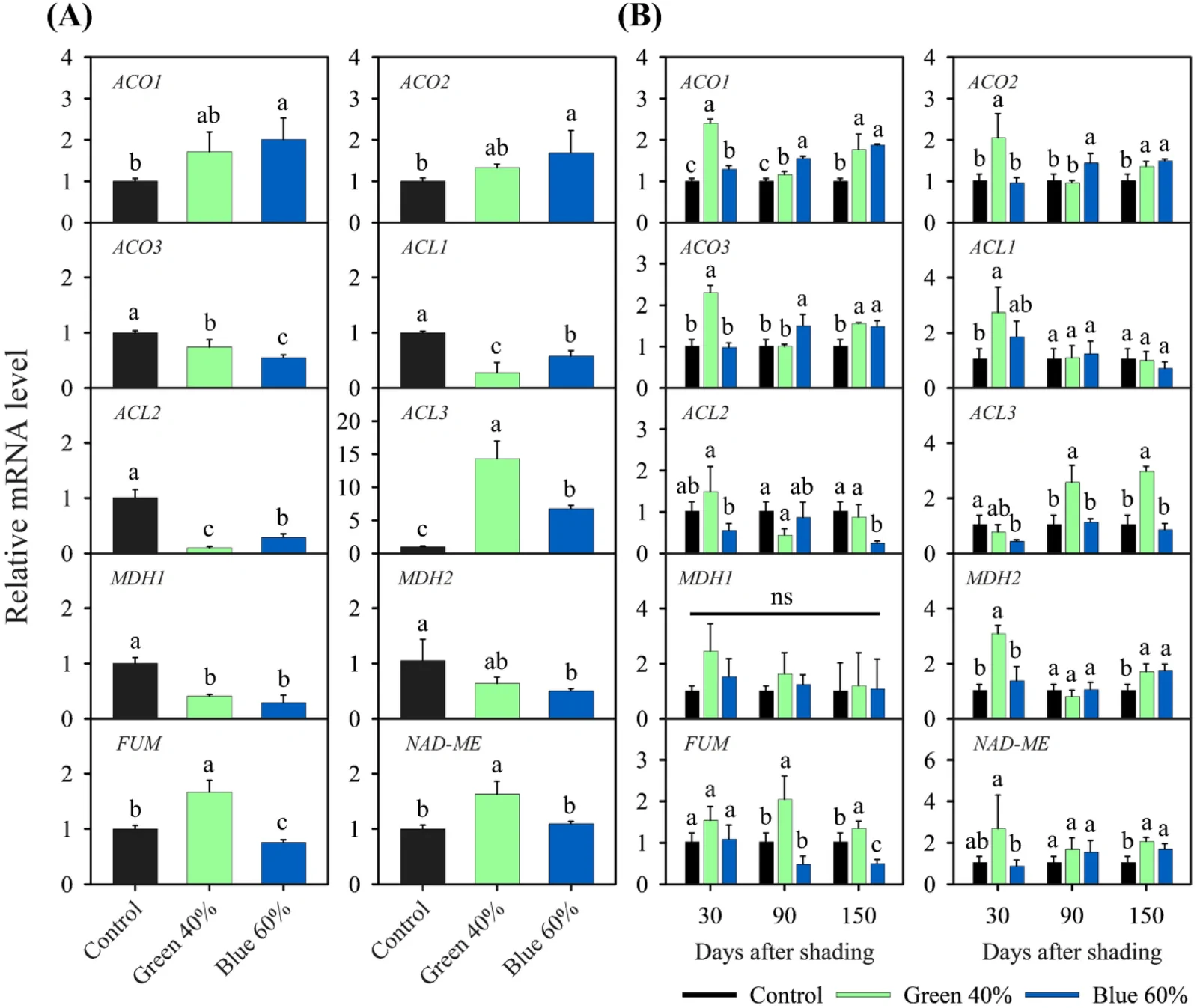
Transcript comparison of citrate-malate degradation-related genes under color shading nets during the field experiments of 2023–2024 at harvest **(A)** and 2024–2025 at three different stages after shading **(B)**. Values are the mean ± SD. A column with different lowercase letters indicates a statistically significant difference (P< 0.05) when the data were analyzed by analysis of variance, followed by Duncan’s multiple range tests.

### 3.7 Transcript analysis of citrate-malate transportation-related genes

During the 2023–2024 shading season (**Figure 9A**), the strongest treatment effect was on *CsPH8*, which was highest under blue 60%, intermediate in the control, and lowest under green 40%, indicating that blue shading enhanced the tonoplast proton-pumping capacity that supports vacuolar acidification and organic-acid sequestration; *ALMT9* showed no significant change among treatments, suggesting a limited role for this transporter in explaining acidity differences at that stage, while *ALMT9-LIKE* was suppressed under green 40% but remained comparable between the control and blue 60%, and *CsCIT1* was highest under green 40%.

**Figure 9:**
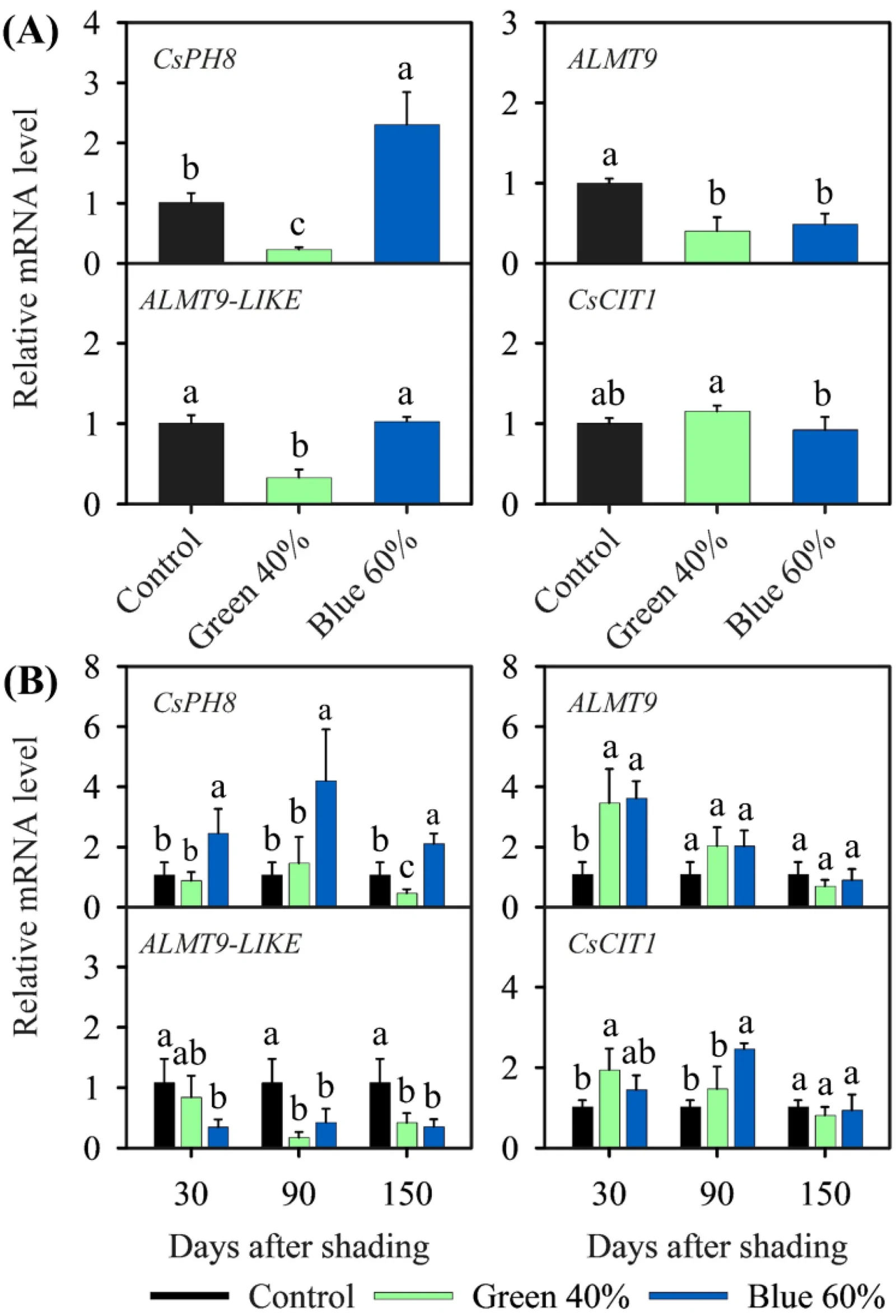
Transcript comparison of citrate-malate transportation-related genes under color shading nets during the field experiments of 2023–2024 at harvest **(A)** and 2024–2025 at three different stages after shading **(B)**. Values are the mean ± SD. A column with different lowercase letters indicates a statistically significant difference (P< 0.05) when the data were analyzed by analysis of variance, followed by Duncan’s multiple range tests.

In the 2024–2025 shading season (**Figure 9B**), regulation was clearly stage-dependent: *CsPH8* was consistently and significantly upregulated under blue 60% at 30, 90, and 150 DAS, supporting a sustained increase in vacuolar proton gradient formation and therefore a greater potential for acid storage/retention under shading; *ALMT9* again varied only modestly without clear significance, whereas *ALMT9-LIKE* tended to be lower under both shade nets (especially blue 60%) across the season, and *CsCIT1* showed transient peaks higher under green 40% at 30 DAS and under blue 60% at 90 DAS before converging later. Overall, blue shading 60% enhances acidity largely by increasing vacuolar acidification (*CsPH8*) and temporally regulating citrate transport, instead of consistently inducing malate transport through *ALMT9*.

### 3.8 Clustered analysis of gene expression

The heatmap analysis across the three ripening stages indicates that colored shade nets reprogrammed organic-acid metabolism through coordinated, pathway-level shifts rather than uniform changes in single genes (**Figure 10**). A main feature is the contrasting regulation of acid storage/synthesis modules versus acid breakdown modules: the blue 60% shade net shows stronger expression of genes associated with citrate accumulation and vacuolar sequestration, most notably the tonoplast proton pump *CsPH8* (and the citrate-synthesis component *CS2*/*CS1*), particularly at mid-to-late stages, which would enhance vacuolar acidification and promote citrate retention as fruit ripen. In contrast, green 40% displays relatively higher expression of several degradation-associated genes, including *ACO, ACL, MDH2, NAD-ME*, and *FUM* in clustered catabolic groups, especially at earlier stages. Overall, the heatmap reveals contrasting transcriptional patterns: blue 60% shows stronger expression of genes associated with acid synthesis and vacuolar sequestration, whereas green 40% shows higher expression of several degradation-associated genes, including *NAD-ME*. These transcript patterns are consistent with the contrasting acidity phenotypes but do not establish corresponding changes in enzyme activity or metabolic flux.

**Figure 10:**
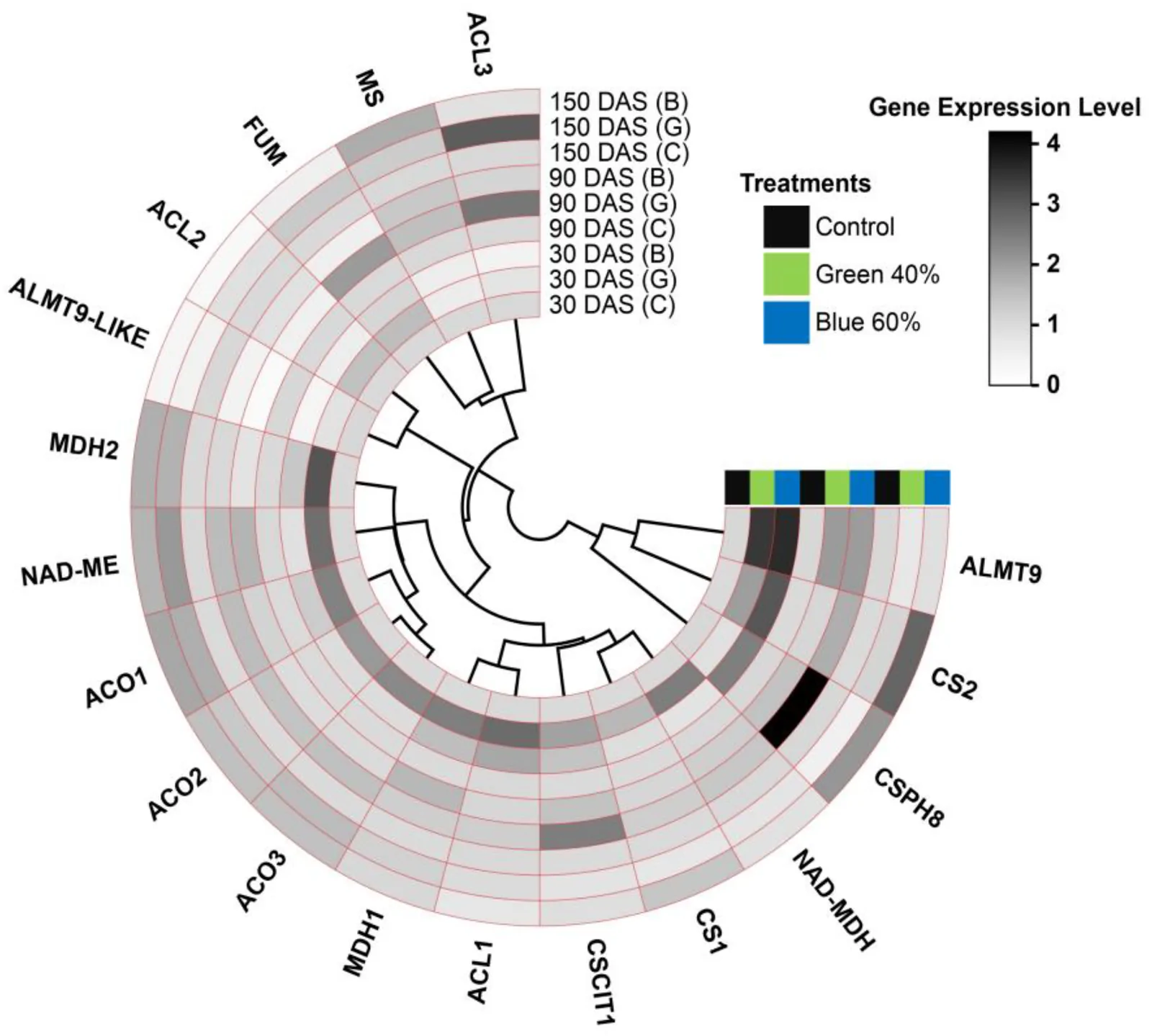
Heatmap of organic acid-related genes at three different ripening stages under color shade nets compared to the open field during the field experiment (2024-2025). Expression levels: dark color (high), light color (low).

### 3.9 Correlation between all parameters and organic acid genes

Pearson correlation analysis revealed two contrasting clusters (**Figure 11**). PAR, LUX, and temperature clustered with ABA, proline, CAT, and POD and showed predominantly negative correlations with *Fv/Fm* and fruit organic-acid traits. In contrast, RH and *Fv/Fm* were positively correlated with citric acid, malic acid, and titratable acidity and clustered with *CS2*, *CS1*, and CsPH8; *CsCIT1* was also associated with the acidity-related cluster. *ACO* isoforms, *ACL3*, and *FUM* clustered with the higher-PAR/higher-temperature variables and showed opposite relationships with acidity.

**Figure 11:**
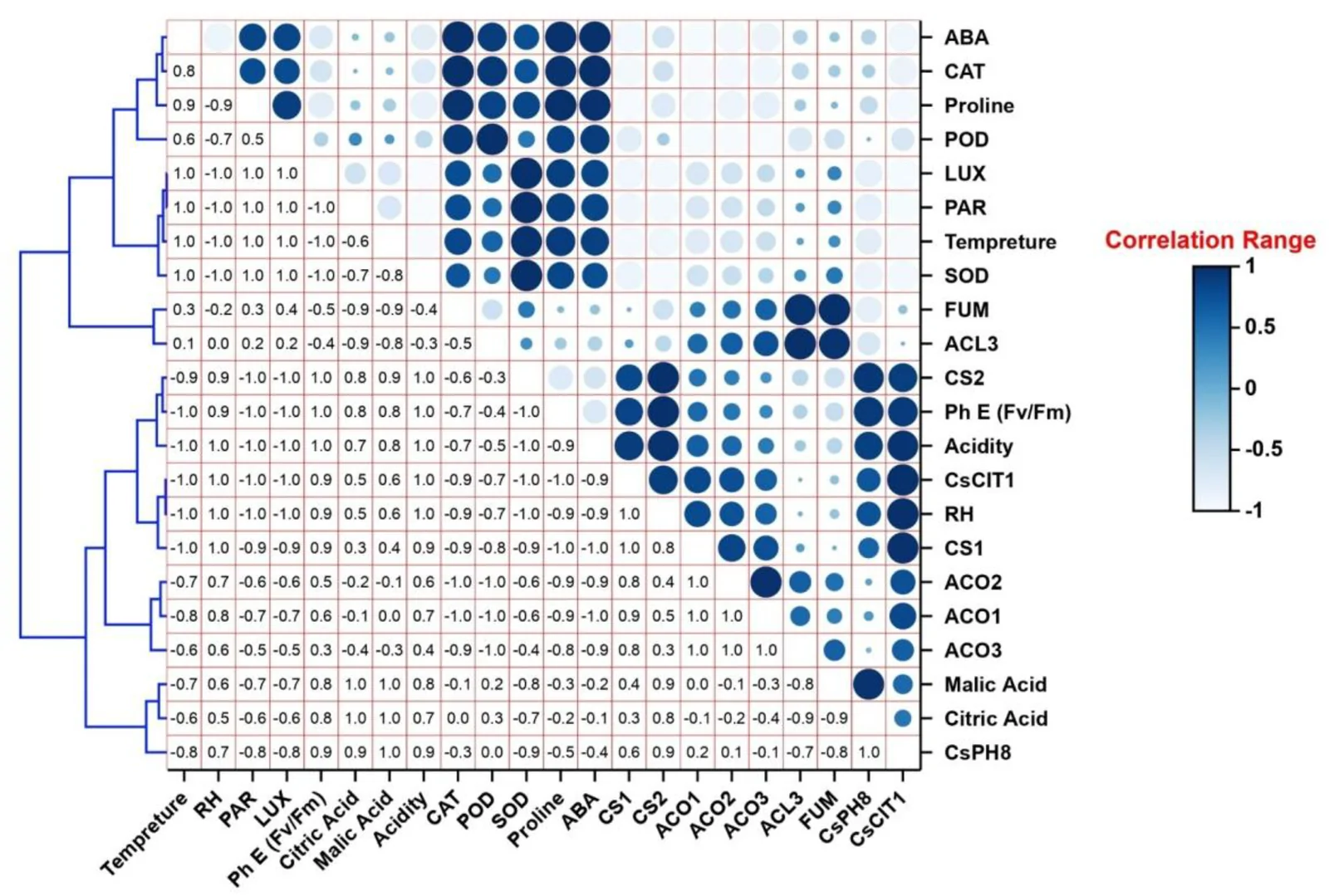
Pearson correlation analysis between environmental and photometrics parameters, physiological measurements including organic acids and acidity, and differentially expressed genes (DEGs) related to synthesis, degradation, and transport of organic acids during shading season 2024-2025. Dark colors indicate strong positive correlations, and light colors indicate strong negative correlations.

## 4 Discussion

Colored shade nets modified both light quantity and spectral quality, but these effects should be interpreted separately. In the first season, mean temperature and relative humidity (RH) were influenced mainly by shading intensity: 60% nets produced greater reductions in temperature and greater increases in RH than 40% nets, whereas net color had no significant effect on these two microclimatic variables. PAR and light intensity were also lower under 60% than 40% shading. Therefore, the general improvement in PSII performance (*Fv/Fm*) and the reduction in stress-associated responses under shading are more conservatively attributed primarily to reduced irradiance and heat load and to microclimate moderation rather than to a direct effect of net color (Kabir et al., 2024; Elshahat et al., 2025; Yin et al., 2025). This interpretation is consistent with the sensitivity of PSII to excessive irradiance and elevated temperature, both of which can promote photoinhibition and reduce *Fv/Fm* under field conditions (Baker, 2008; Sembada et al., 2024; Zarrin Ghalami et al., 2026).

The combined *Fv/Fm*, antioxidant-enzyme, proline, and ABA responses provide a broader indication of plant stress status under the different light environments. Shaded treatments maintained or slightly improved *Fv/Fm* compared with the open field and generally showed lower CAT and POD activities, proline accumulation, and ABA levels, whereas SOD activity showed no consistent significant treatment response. Increased CAT and POD activities are commonly associated with greater demand for H_2_O_2_ detoxification during abiotic stress, while ABA and proline participate in stress signaling, osmotic adjustment, and redox homeostasis (Gill and Tuteja, 2010; Szabados and Savouré, 2010; Vishwakarma et al., 2017). Because reactive oxygen species and direct markers of oxidative damage were not measured in the present study, the antioxidant-enzyme responses should be interpreted as reflecting differences in antioxidant demand rather than as direct evidence of oxidative injury. Overall, the physiological patterns are consistent with lower photochemical and thermal stress under shade.

Importantly, however, improved physiological stress status under shading did not by itself explain the contrasting effects on fruit acidity. Both blue 60% and green 40% generally reduced several stress-associated indicators relative to the open-field control, yet they produced opposite organic-acid phenotypes. Blue 60% maintained the highest citric acid, malic acid, and titratable acidity during fruit development, whereas green 40% consistently showed the lowest values. This divergence suggests that microclimate moderation and maintenance of PSII function represent a common response to shading, whereas the treatment-specific differences in organic-acid retention were additionally associated with metabolic and transcriptional responses. Final fruit acidity reflects the balance among organic-acid synthesis, degradation, and vacuolar compartmentation, all of which vary during fruit development and can respond to environmental conditions (Mao et al., 2024; Su et al., 2025). The observed correlations between higher PAR, LUX, and temperature and lower organic-acid traits, together with the positive associations of RH and *Fv/Fm* with acidity, are consistent with this interpretation. Nevertheless, these correlations do not establish causality.

At the molecular level, the transcriptional responses could be grouped broadly into genes associated with citrate synthesis (*CS1* and *CS2*), citrate/malate utilization and degradation (*ACO*, *ACL*, *MDH*, *NAD-ME*, and *FUM*), and vacuolar acidification and transport (*CsPH8*, *CsCIT1*, and related transporters) (Casal and Yanovsky, 2005; Morciano et al., 2019; Zha et al., 2022). Blue 60% showed sustained higher *CsPH8* transcript abundance together with stage-specific increases in *CS2* and *CS1*, whereas green 40% showed higher expression of several degradation-associated genes, including *ACO* isoforms, *ACL3*, *FUM*, and *NAD-ME*. These contrasting transcription patterns were consistent with the corresponding high- and low-acidity phenotypes, but they should not be interpreted as direct evidence of changes in enzyme activity or metabolic flux.

*NAD-ME* catalyzes the oxidative decarboxylation of malate to pyruvate and therefore provides a potential route for malate utilization. In the present study, higher *NAD-ME* transcript abundance under green 40% coincided with lower malic acid accumulation, suggesting a possible contribution to greater malate utilization. However, because *NAD-ME* enzyme activity and metabolic flux were not measured, this relationship remains associated. Das et al. (2025) reported that combined H_2_S and blue-light treatment affected malic acid metabolism in postharvest pepper fruit and implicated *NADP-ME* in this response. Because that study examined *NADP-ME* rather than *NAD-ME*, and in a different species and postharvest context, it provides broader evidence that light-related treatments can influence malate metabolism but not direct functional evidence for the *NAD-ME* response observed here.

Among the vacuolar-associated genes, *CsPH8* showed the clearest relationship with the high-acidity phenotype under blue 60%. Previous studies have identified *CsPH8* as an important regulator of vacuolar acidification and citrus fruit acidity (Shi et al., 2019; Alam et al., 2022). The stage-dependent changes in *CsCIT1* were also consistent with its established role in citrate transport into the vacuole (Shimada et al., 2006; Sweetman et al., 2009; Zhang et al., 2024). Accordingly, the higher acidity under blue 60% was associated with a transcriptional pattern involving acid synthesis and vacuolar acidification/storage, whereas the lower acidity under green 40% was associated with greater expression of several degradation-related genes. These associations provide a plausible framework for interpreting the contrasting acidity responses but do not demonstrate that any individual gene directly caused the observed changes in organic-acid accumulation.

The correlation analysis further integrates the physiological and molecular responses. Higher PAR, LUX, and temperature were associated with higher ABA, proline, CAT, and POD responses and with lower *Fv/Fm* and organic-acid traits, whereas higher RH and *Fv/Fm* were associated with greater citric acid, malic acid, titratable acidity and with expression of several acid synthesis- and storage-related genes. Thus, the overall pattern is consistent with an association among microclimate moderation, maintained PSII function, reduced stress responses, and greater organic-acid retention.

Overall, the present results indicate that colored shade nets can modify orchard microclimate, physiological stress responses, and fruit organic-acid metabolism. The physiological responses appear to be associated mainly with reduced radiation and heat load, whereas the contrasting acidity phenotypes of blue 60% and green 40% were additionally associated with distinct developmental patterns of organic-acid-related gene expression. Future studies combining matched shading intensities with spectral measurements, enzyme-activity assays, and metabolic-flux analyses will be necessary to distinguish the relative contributions of light quality, shading intensity, and microclimate more directly. The proposed model summarizing the associations among shade-induced microclimatic changes, physiological responses, organic acid-related gene expression, and fruit acidity under blue 60% and green 40% shading is presented in **Figure 12**.

**Figure 12:**
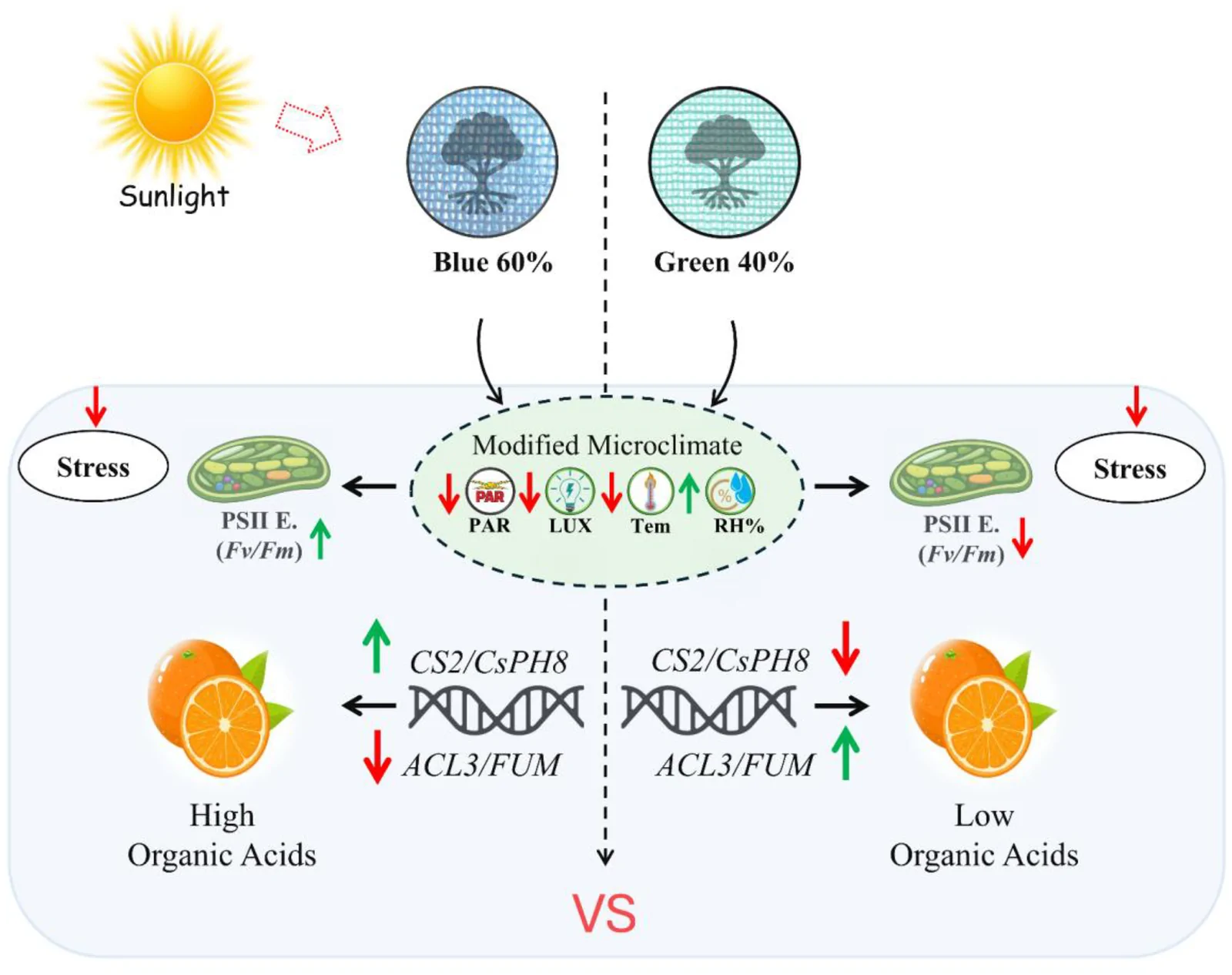
A model illustrates the impact of blue 60% and green 40% shade nets on organic acid accumulation in citrus fruit by regulating the microclimate around the trees and enhancing PSII E. Therefore, its effect on genes is related to organic acid. Red arrows signify a decrease in levels, while green arrows indicate an increase.

From a practical perspective, total yield did not differ significantly between the open-field and shading treatments, possibly because shading was initiated after full flowering and fruit set. Thus, colored shade nets may be particularly relevant for high-value and export-oriented citrus production, where maintaining fruit acidity under warm, high-radiation conditions could provide an additional commercial advantage. However, the present study did not assess soluble sugars, vitamin C, commercial fruit rate, or the costs of net installation and maintenance. Future multi-cultivar and multi-location trials should therefore integrate agronomic performance, broader fruit-quality attributes, and cost-benefit analyses to determine the economic feasibility of colored shade nets for commercial citrus production.

## 5 Conclusions

Across two growing seasons, colored shade nets moderated the orchard microclimate by reducing PAR, light intensity, and canopy temperature while increasing relative humidity. These changes were accompanied by maintained or slightly improved PSII efficiency (*Fv/Fm*) and generally lower stress-associated responses, including ABA, proline, and CAT/POD activities, compared with the open-field control. Among the treatments evaluated, blue 60% most consistently maintained higher citric acid, malic acid, and titratable acidity during fruit development, whereas green 40% produced the lowest acidity. The contrasting acidity phenotypes were associated with distinct transcriptional patterns: blue 60% showed sustained higher *CsPH8* expression and stage-specific increases in *CS2* and *CS1*, while green 40% showed higher expression of several degradation-associated genes, including *ACO* isoforms, *ACL3*, *FUM*, and *NAD-ME*. These transcriptional responses should be interpreted as associations because corresponding enzyme activities and metabolic fluxes were not directly measured. Moreover, because blue 60% and green 40% differed in both net color and shading intensity, their comparison cannot isolate a direct spectral effect. Nevertheless, among the tested treatments, blue 60% represents a promising candidate for maintaining citrus fruit acidity while moderating environmental stress under warm, high-radiation orchard conditions.

## Data Availability

The datasets presented in this study can be found in online repositories. The names of the repository/repositories and accession number(s) can be found in the article/**Supplementary Material**.
