# Supplementary material for: Blue and green shade nets differentially regulate organic acid accumulation in citrus fruit
Source: Front Plant Sci. 2026 Oct 14;17:1969799. doi: 10.3389/fpls.2026.1969799 (PMC13713395; doi:10.3389/fpls.2026.1969799)
Supplement: Supplementary file 1 [file DataSheet1.docx]

**Fig. S1.** Some fruit characteristics from fruit samples in the open field treatment compared to those from various shading treatments during the 2023/2024 season.
