# Supplementary material for: Blue and green shade nets differentially regulate organic acid accumulation in citrus fruit
Source: Front Plant Sci. 2026 Oct 14;17:1969799. doi: 10.3389/fpls.2026.1969799 (PMC13713395; doi:10.3389/fpls.2026.1969799)
Supplement: Supplementary file 2 [file Table1.docx]

| **Table S1: Citrate-Malate metabolism-related genes** | | | | | | |
| --- | --- | --- | --- | --- | --- | --- |
| 1 | Synthesis | CS1 | Citrus synthase | CTCACTGGATTATGGTGGGAACT | GCACTGACATTTCCACCTTCG | Cs7g01170 |
| 2 |  | CS2 |  | CGAAACAAGTGAGGGACAAAC | GAAGAGAACATCCAGCACACG | orange1.1t01588 |
| 3 |  | cytNAD-MDH | Cytosolic NAD depended malate dehydrogenase | ATTCGTGGCGGAAGTTTTG | GCGGTTCGTCAGGTATTCAGT | Cs8g03230 |
| 4 |  | MS | Malate synthase | TGCCCACTATGATGTCCCTG | TCCCTACGACACTCCATTGC | Cs5g06930 |
| 5 | Degradation | ACO1 | Aconitate hydrates | ACAGCCTATGAGCATGGTCTTG | TTGGCAATGGTAGCACGGT | Cs1g26040 |
| 6 |  | ACO2 |  | TACCTTGGACGGGTTGTCTTTA | TTCCTCCAACTCCCCAACC | Cs4g10280 |
| 7 |  | ACO3 |  | TGGAAACAGGAATTTTGAGGG | TCCAATCGGCTCCTTATCAA | Cs2g21430 |
| 8 |  | ACL1 | ATP-citrate lyase | AGAGTGGTTTGGTGGCATTG | CATCCAAGCCTATCGGAGACTA | Cs6g01210 |
| 9 |  | ACL2 |  | ATGGAGATGAGTGGATGTAAAGG | GCACCTGTTGGGACAAAGATA | Cs7g08950 |
| 10 |  | ACL3 |  | AGGAGATGTGTTCCCTGGCT | GGTTTGTTTACCTTTCCCTGTT | Cs9g02230 |
| 11 |  | MDH1 | Malate dehydrogenase | GCCATTCACATTGTTAGCGTCC | CACCAAGTAGCGGCGGAAT | Cs1g25560 |
| 12 |  | MDH2 |  | TGCCCCATCGCATCTTCT | CCCAACCAAAGCCTAAAACCT | orange1.1t01255 |
| 13 |  | FUM | Fumarate hydrates | TCCGATAAACTGTGGGGGG | GGCAGCACATTTCTTGAGGAT | Cs5g33580 |
| 14 |  | NAD-ME | NAD dependent Malic enzyme | GGGGAGAAAGTCCTCATACAGTT | CTCCAGCACCCAGGAACAA | Cs4g19200 |
| 15 | Transportation | CsPH8 | P-type proton pump | CCGTGAAGGAATTGATTTGG | CCATGACAATGGATTCCACA | Cs1g16150 |
| 16 |  | ALMT9-like | Aluminum activated malate transporter | AATGACCAACAAGCCGATGC | CACCCAATCCTCCGTCATCT | Cs5g02830 |
| 17 |  | ALMT9 |  | GAGGGCTTGCTCTTGCTATG | ATAAGGCTTCATCGTCGGGTA | Cs7g26550 |
| 18 |  | CsCit1 | Citrate symporter, tonoplast dicarboxylic transporter | GACACCGTTGCTCATTCTTACAT | GGCGGATTCATTGGCTCT | Cs2g02120 |
| Actin | | | | CCGACCGTATGAGCAAGGAAA | TTCCTGTGGACAATGGATGGA | XM_006464503 |
